# Revulsion

**Published:** 1869-04

**Authors:** J. Haines

**Affiliations:** Keokuk, Iowa


THE
### IOWA MEDICAL JOURNAL.

VOL. V. *March and April*, 1869. No. 5.

### REVULSION.

BY J. HAINES, M.D , KEOKUK, IOWA.

THE human body being endowed by nature with an organism for the elimination of foreign or poisonous properties from the system, by means of which a correct standard of health is maintained, it must be apparent that whenever this nicely adjusted balance of forces in the human system is impaired or destroyed, functional derangement and organic disease ensue.

All pathologists are familiar with the not unfrequent efforts on the part of nature to restore this lost balance of vital forces by vicarious issues, for the relief of some vital organ — such as the enlargement and opening of the hemorrhoidal veins, fistula in ano, chronic ulcers in the lower extremities, etc., etc.

Endeavoring to follow the unerring lights of nature, we present, for the consideration of the profession, the following:

Oct. 18, 1868 — Called on Mr. H——, aged 55, at his place of business. Observed that he was subjected to frequent paroxysms of coughing, and expectorating a frothy mucus, mixed with pus; said he felt well, aside from the annoyance of his frequent coughing, and would not brook the idea that he was seriously ill.

Nov. 2 — Called again on Mr. H. on business. Found him troubled with frequent paroxysms of coughing, and expectorating more or less pus on every such effort; his features presenting a pale, depressed expression; again I endeavored to impress upon his mind a true sense of his diseased condition and danger, but with so little effect, that I only gained his consent to use a simple expectorant of the acetate of squills.

Nov. 16 — Called to see Mr. H. at his residence. Found him quite ill; pulse 136; dry, hacking cough; complained of sharp pain in the lower portion of the right lung; skin dry and hot. Veratim was administered at intervals of about two hours, until the pulse was reduced to about eighty per minute, and free diaphoresis established — requiring a space of time not exceeding eight hours — when the further use of the veratrum was dispensed with; the pulse never after getting over ninety, and for the greater part of his protracted illness not exceeding seventy-five, and quite feeble.

Immediately following the control of the heart's action and relaxation of the skin, expectoration became free, and large quantities of vitiated pus was discharged. A supporting treatment was instituted, consisting Tr. Ferri. Mur. and quinine, together with the frequent use of stimulants and a good nourishing diet. Despite of every effort to sustain and brace up the vital powers, it became apparent, from day to day, that the progress of the disease was unchecked.

On the 23d of November, I requested Prof. J. C. Hughes to see the case with me, who, after a full and careful examination, recommended a continuation of the treatment, not feeling at liberty, as he said to offer any encouragement as to the final result.

Nov. 25.—Fully two-thirds of the right lung having perished, and the recuperative powers of nature seeming powerless to arrest its further progress, it was apparent that unless some vicarious agent or force could be called into action, the life forces must finally and speedily yield. Under this discouraging aspect of the case, I determined to adopt that principle of action in the future management of the case, so often observed in the spontaneous action of nature, in instituting or establishing a vicarious issue for the protection or relief of some more vital part. In pursuance of this idea, the introduction of a seaton was proposed. But Mr. H. objecting to this, a large blister was substituted, and drawn on the right side of the chest, a little below the line of diseased action. Within twelve hours from the time the blister was drawn, the destruction of the lung seemed to be arrested; and whenever thereafter the blistered surface was allowed to close—which, from inattention on the part of the nurse, was permitted to occur twice or thrice — almost immediately indications of further destruction of the lung would become manifest; but was as speedily arrested by the renewal of the blister. This treatment was continued, Mr. H. daily mending, up to Feb. 2, when my visits were discontinued.

Feb. 10, 1869.—Saw Mr. H. at his place of business, full half a mile from his residence, whither he had walked; says he feels quite well, except that he is not so strong as formerly; but feels that he is gaining in flesh and strength daily.

Jan. 9, 1869.—Called to see Mr. B——, aged about 45. On examination, found but slight febrile excitement, mental faculties clear, frequent small evacuations from the bowels, always mingled with pus, and not unfrequently stained with fresh blood; occasionally from one to three ounces of quite offensive pus, slightly stained with blood, would be discharged, free from the presence of any fecal matter; great tenderness, on pressure, existed throughout the entire gastric and alementary canal, accompanied with frequent vomiting of small quantities of blood from the stomach. The pathological indications seemed to be that of chronic ulceration of the entire glandular structure of the alementary canal. Over the region of the transverse colon there appeared to be an elevation of about half an inch, and of a doughy resistance; this portion of the bowel I supposed to be the original seat of diseased action.

This case had occasionally engaged my attention for over two months prior to this examination, and for which I had prescribed a palative treatment, not being able to subject him to anything like a thorough management, owing to his indomitable energy and the urgency of business.

The following treatment was instituted: constant rest in a a horizontal posture, the entire surface of the body to be thoroughly bathed or sponged twice daily with spirits—good whisky being used; mucillaginous drinks, with boiled milk, thickened with a little flour, as a diet, taken frequently in small quantities; injections of thin starch with the Tr. Opii and Acetatis Plumbi, given *pro re nati*, to control the peristaltic action of the bowels. As an alterative, the following prescription was given, and repeated at intervals for several days:

| | | | |
|---|---|---|---|
| ℞. Calomel, | | gr.vii. | Made into fine powders and given at four hours intervals, to be followed by a dose of castor oil, some five or six hours after the powders were taken. |
| Ipecac, | pulv. | gr.iss. | |
| Gum Myrhh, | " | gr.iij. | |
| Opii, | " | gr.iij. | |
| Gum Acacia, | " | scrup.iss. | |

Sp. Turpentine, in doses of from ten to twelve drops, were given in intervals of five hours, throughout the entire treatment of the case.

Jan'y 15.—Not being able to discern any improvement in the symptoms or general condition of Mr. B., a blister was ordered, covering the entire extent of the transverse colon. Soon after this blister was drawn, a general improvement beacme manifest, and continued, *pari pusue*, with the repeated renewal of the blister; and when, from neglect, the blistered surface was allowed to heal, as did occur some twice or thrice, all the symptoms assumed a more malignant type.

During this treatment of the case, all medicines except the turpentine, and the injections, as before stated, were discontinued. The bowels were cleansed daily, by the administration of small doses of castor oil.

Under this treatment Mr. B. continued to improve, and was discharged convalescent, Feb. 7th, 1869.

